# Comparison between Appendicular Skeletal Muscle Index DXA Defined by EWGSOP1 and 2 versus BIA Tengvall Criteria among Older People Admitted to the Post-Acute Geriatric Care Unit in Italy

**DOI:** 10.3390/nu12061818

**Published:** 2020-06-18

**Authors:** Sawsan Hilal, Simone Perna, Clara Gasparri, Tariq A. Alalwan, Viviana Vecchio, Federica Fossari, Gabriella Peroni, Antonella Riva, Giovanna Petrangolini, Mariangela Rondanelli

**Affiliations:** 1 Department of Mathematics, College of Science, University of Bahrain, Sakhir Campus, P.O. Box 32038 Zallaq, Bahrain; shilal@uob.edu.bh; 2 Department of Biology, College of Science, University of Bahrain, Sakhir Campus, P.O. Box 32038 Zallaq, Bahrain; sperna@uob.edu.bh (S.P.); talalwan@uob.edu.bh (T.A.A.); 3 Endocrinology and Nutrition Unit, Azienda di Servizi alla Persona ‘‘Istituto Santa Margherita’’, University of Pavia, 27100 Pavia, Italy; viviana.vecchio01@universitadipavia.it (V.V.); federica.fossari01@universitadipavia.it (F.F.); gabriella.peroni01@universitadipavia.it (G.P.); 4 Research and Development Unit, Indena, 20139 Milan, Italy; antonella.riva@indena.com (A.R.); giovanna.petrangolini@indena.com (G.P.); 5 IRCCS Mondino Foundation, 27100 Pavia, Italy; mariangela.rondanelli@unipv.it; 6 Department of Public Health, Experimental and Forensic Medicine, University of Pavia, 27100 Pavia, Italy

**Keywords:** sarcopenia, appendicular skeletal muscle, appendicular skeletal muscle index, prevalence, elderly

## Abstract

This study aims to assess the agreement between the appendicular skeletal muscle index (ASMI) and dual-energy X-ray absorptiometry (DXA) using a single frequency bioelectrical impedance analysis (BIA) to assess criteria. Moreover, we used the European working group on sarcopenia in older people 1 (EWGSOP1), EWGSOP2, and the Tengvall equation to estimate a low prevalence in ASMI (under the cutoff criteria). We examined a sample of 765 elderly individuals (27.8% male and 72.2% female, aged 82 ± 8.2 years). Based on the cutoff identified by Tengvall, EWGSOP1, and EWGSOP2, the results showed that the prevalence of low ASMI in females was 10.1%, 11.4%, and 9.2%, respectively, and 98.1%, 30.5%, and 23.5% in males, respectively. Moreover, low ASMI prevalence under each diagnostic criterion and body mass index (BMI) was calculated. For BMI < 25 kg/m^2^, the ASMI prevalence was 39.9%, 25.9%, and 20.6%, as determined using Tengvall, EWGSOP1, and EWGSOP2, respectively, and for BMI > 25 kg/m^2^, the ASMI prevalence was 29.0%, 6.6%, and 5.2%. The percentage of agreement and Cohen’s Kappa with the corresponding *p*-value between Tengvall and EWGSOP1 was 70.1% (*p* < 0.001). Between Tengvall and EWGSOP2, it was 69.4% (*p* < 0.001). Between EWGSOP1 and EWGSOP2, it was 96.5% (*p* < 0.001). Regarding gender, low ASMI prevalence in males was higher than in females. Moreover, in females, the prevalence was comparable among the three diagnostic criteria, while in males, it was significantly higher under Tengvall than the other two criteria. The application of the Tengvall formula with a single frequency BIA should be revised in terms of application for assessing low ASMI in elderly patients.

## 1. Introduction 

Sarcopenia is a progressive and generalized skeletal muscle mass disorder that occurs most frequently in the elderly. Sarcopenia is associated with an increased risk of adverse outcomes, including falls, fractures, physical disability, and mortality [1]. Baumgartner gave the first operational definition, providing a method for estimating the prevalence of lean body mass, using appendicular skeletal muscle mass (ASM) that was adjusted for height (kg/m^2^) [2]. The estimation of the low appendicular skeletal muscle mass index (ASMI) varies, reflecting the different approaches to its definition. The most recent update on ASMI was carried out in 2018 by the European working group on sarcopenia in older people 2 (EWGSOP2) [3] (<5.5 kg/m^2^ for women, <7.0 kg/m^2^ for men) [4]. Using this diagnostic criterion, compared to the previous one (EWGSOP1) [5] whose diagnosis was based on the documentation of low muscle mass, the prevalence data decreased as a result of the algorithm change and its thresholds [6,7,8]. Formulas for the diagnosis of low ASMI are well defined according to EWGSOP1 and EWGSOP2 [9] criteria.

Dual-energy X-ray absorptiometry (DXA) provide useful data for ASMI. According to equations developed by Tengvall [10], Jansenn [11], and Kyle [12], even bioelectrical impedance analysis (BIA) and relative formulas for lean mass calculation could be interesting for muscle evaluation. BIA data allow for the calculation of relative skeletal muscle mass index (RSMI). 

Although the diagnosis of lean body mass (LBM) loss must follow the cutoff proposed by EWGSOP2, the execution of this algorithm is difficult to perform in everyday clinical practice. Thus, simple, reproducible, and cost-effective techniques are necessary for an early diagnosis of LBM loss in older patients in everyday clinical practice. Therefore, the primary outcome of this paper was to assess if an agreement exists between EWGSOP2, ASMI, and other criteria, such as the Tengvall Equation. The secondary outcome was to estimate LBM loss according to these ASMI criteria and to consider associated risk factors.

## 2. Materials and Methods 

### 2.1. Study Design

A cross-sectional study was carried out among elderly individuals living in Pavia, Italy. The inclusion criteria included Caucasian patients aged 65 years or older who were either admitted to the post-acute geriatric care unit for functional secondary loss to a nondisabling medical disease, were bedridden, were ambulatory prior to hospitalization, and were willing to participate by signing the informed consent form after receiving information about the study. The study was approved by the Department of Internal Medicine and Medical Therapy at the University of Pavia ethics committee, with code 6723/22052019.

The patients were not diagnosed as having disabling conditions that could directly affect muscle weakness (i.e., neurological diseases, hip fractures, or amputations) at the time of admission. However, exclusion criteria for the study included patients with diabetes, rheumatological disease, metabolic disease, or neoplasia. Exclusion also pertained to those treated with steroids, statins, and those unable to walk.

### 2.2. Participants

Elderly men and women were evaluated after admission to the geriatric physical medicine and rehabilitation division at the Santa Margherita Hospital, Azienda di Servizi alla Persona of Pavia in Pavia, Italy. Every subject underwent complete medical screening before participation. This included vital signs, urine tests, blood tests, and a 12-lead electrocardiogram.

### 2.3. Assessment of Anthropometric Parameters and Body Composition

Body weight was measured to the nearest 0.1 kg on an electronic scale, with subjects wearing light clothing and no shoes using a standardized method [13]. Waist measurements were taken at the midpoint between the lowest rib and the top of the hip bone (iliac crest), with the use of a standardized technique [13].

Body composition (i.e., LBM, fat mass, and gynoid and android fat distribution) was measured using DXA with the use of a Lunar Prodigy DXA (GE Medical Systems, Chicago, IL, USA). The in vivo CVs were 0.89% and 0.48% for whole body fat (fat mass) and LBM, respectively. The relative skeletal muscle mass (RSMM) was taken as the sum of the skeletal muscle of arms and legs [14].

The state of hydration for elderly subjects was assessed with BIA, since changes in fluid status would affect the soft tissue composition estimated using DXA [15,16]. Measurements of whole-body resistance and reactance were obtained with the subject supine on a nonconductive table surface using a phase-sensitive, single-frequency impedance plethysmograph (400-mA, 50-kHz alternating current (BIA-101, Akern, Pontassieve, Italy)). Adhesive spot electrodes were placed on the right hand and foot according to the guidelines set forth by the NIH technology assessment conference statement. 

### 2.4. Food Intake Assessment

The mini nutritional assessment (MNA) was used to assess the nutritional status of all participants [17]. This tool uses measurements and a short questionnaire, including an anthropometric assessment of weight, height, and weight loss, in addition to a general assessment (e.g., lifestyle, medication, and mobility), and a dietary assessment (i.e., number of meals, food and fluid intake, self-assessment of autonomy of eating, and self-perception of health and nutrition). The subjects consumed three meals per day.

### 2.5. Handgrip

Muscle function was assessed using the Jamar hand dynamometer (Jamar 503J1, accuracy 0.6 N, Sammons Preston Rolyan, Chicago, IL, USA), via a standardized procedure [18]. 

### 2.6. Health-Related Quality of Life

In order to assess quality of life, participants were subjected to the short-form 36-item health survey (SF-36) [19]. This questionnaire is used for rating health-related quality of life in several fields of research due to its validity, high test–retest reliability, and high internal consistency. Two dimensions were used to summarize the SF-36 scales: “physical health”, comprising the first five scales, and “mental health”, comprising the last five scales. General health scales and vitality are part of both, i.e., each dimension includes three specific and two overlapping scales. Standardized summary scores for mental and physical components were determined and used separately to measure outcomes. The SF-36 survey was conducted before and after the treatment period.

### 2.7. Barthel Index

To evaluate independent living, participants were tested using the Barthel Index (BI) [20], a simple index to score the ability of a patient with a disability disorder related to the functional status and ability to care for himself. By repeating the test periodically, we assessed improvement. This record contains information on how to use it and the entire index.

### 2.8. Criteria Used for Appendicular Skeletal Muscle Index Estimation

- EWGSOP1 ASM/height2 (<7.23 kg/m^2^ for men and <5.67 kg/m^2^ for women);
- EWGSOP2 ASM/height2 (<7.0 kg/m^2^ for men and <5.5 kg/m^2^ for women);
- Tengvall SMI: moderate sarcopenia when SMI is between 8.51 and 10.75 kg/m^2^ (men) or 5.76 and 6.75 kg/m^2^ (women) and severe sarcopenia when SMI is ≤8.50 kg/m^2^ (men) or ≤5.75 kg/m^2^.

### 2.9. Statistical Analysis

The statistical analysis was performed using Statistical Programming Language R version 3.6.0 [21]. The response was a binary variable indicating the presence or absence of a low ASMI based on a certain cutoff where Tengvall, EWGSOP1, and EWGSOP2 were considered. The categorical variables (i.e., smoking status and gender) were summarized in terms of frequency and percentage, while the numerical variables were summarized in terms of the first, second, and third quartiles to account for the non-normality behavior apparently realized in their distributions. The summary statistics were calculated after excluding missing data from the variables of interest. Furthermore, besides the numerical summary, graphical representations were presented whenever appropriate for illustration.

The prevalence of low ASMI was calculated under each diagnostic criterion as an overall estimate calculated using the information of all patients in the study sample as a gender-specific estimate and as a BMI-specific estimate to provide further insights into sarcopenia prevalence within important categories of patients. Moreover, Cohen’s Kappa was used for the agreement analysis among the three diagnostic criteria under consideration. 

To identify risk factors related to muscle loss associated with each diagnostic criterion, we implemented a logistic regression for all patients in the study. However, when the focus was on overweight and obese patients (BMI ≤ 25 kg/m^2^), the significant reduction in sample size after removing missing data hindered the accuracy of the logistic regression. Accordingly, in the latter case, the classification tree was used to identify risk factors. The classification tree is a supervised learning algorithm that gives a graphical representation of the problem in which the predictor space is partitioned following a recursive binary split by starting from the root at the top of the tree then moving to the leaves at the bottom of the tree such that each leaf corresponds to a class label of the response variable. The classification tree is a complex entity to interpret and it is subject to overfitting data, and thus pruning is recommended to focus mainly on the most important factors on which the size of the pruned classification tree is usually specified by the cross-validation approach. Finally, the level of significance was set to 5% for the conducted statistical analysis. 

## 3. Results

### 3.1. Study Sample

The analyzed database consisted of 1068 patients with 64 predictors. However, since one outcome of the current study is the comparison among different cutoffs for ASMI, only patients with complete measurements on the three considered diagnostic criteria were retained, so we ended up with a sample size of 765 patients. Moreover, missing data was an evident feature for many predictors. Accordingly, all predictors missing at least 28% of values were removed from the analysis. The latter adoption resulted in a reduction to 46 predictors. It is worth mentioning that the cutoff point of 28% represents the percentage of missing values for the Tengvall criterion.

### 3.2. Patients’ Characteristics

**Figure A1:**
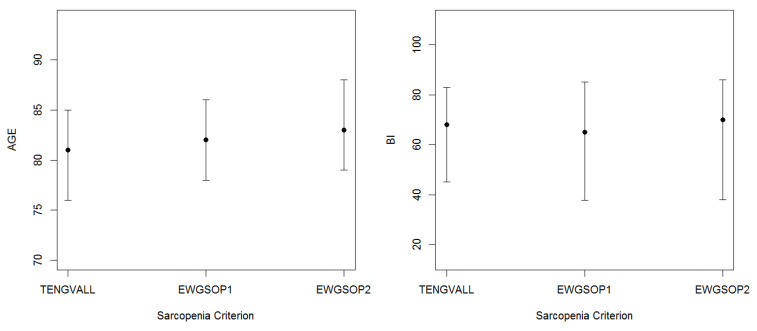
Summary for patients with low ASMI characteristics under each diagnostic criterion. The points represent medians, while lowers are upper quartiles are represented by the vertical bars.

**Figure A2:**
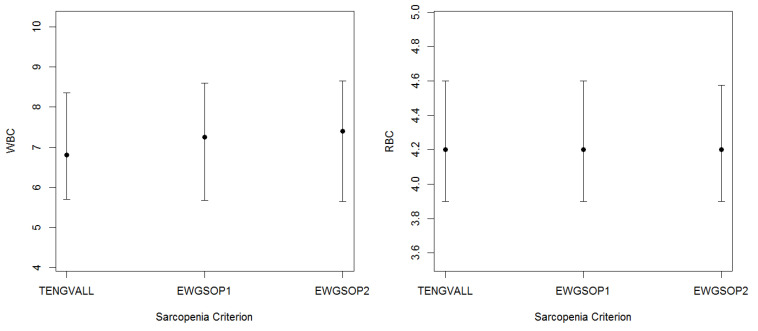

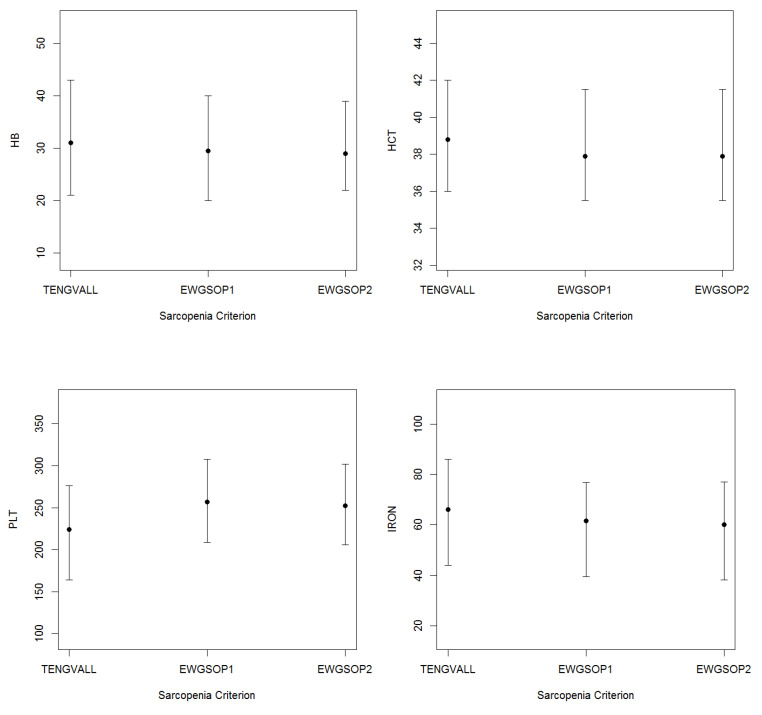
Summary of patients with low ASMI characteristics under each diagnostic criterion. The points represent medians, while lowers are upper quartiles are represented by the vertical bars.

**Figure A3:**
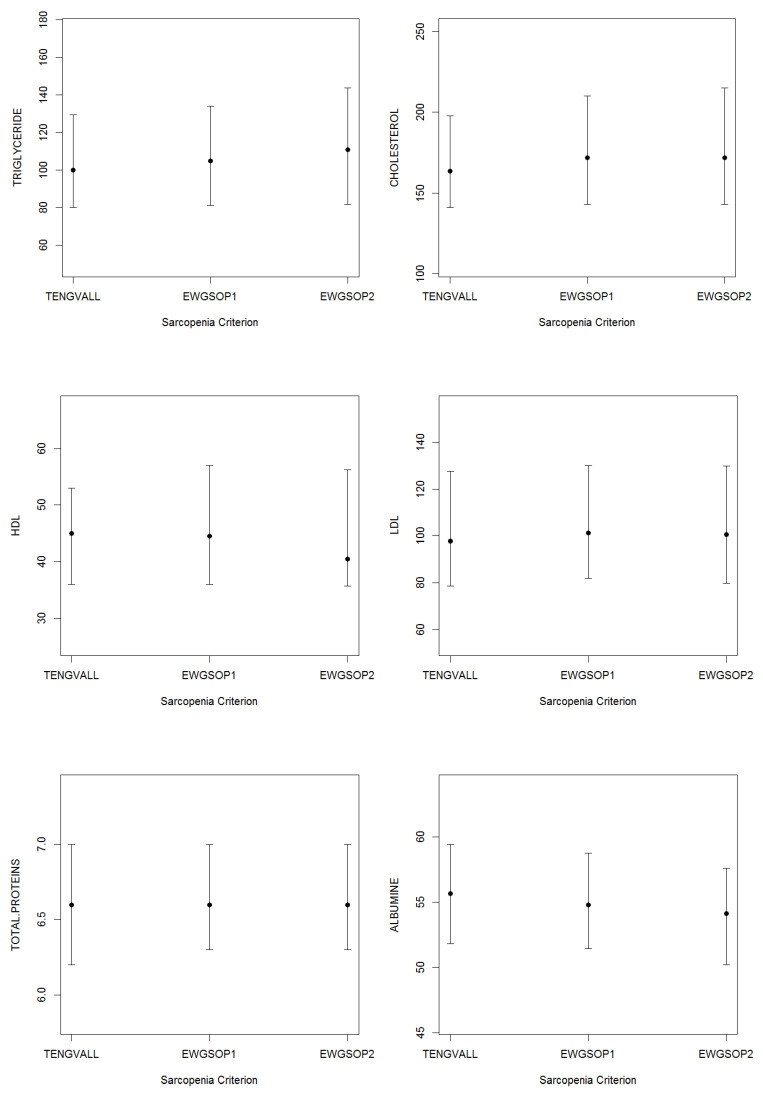
Summary of patients with low ASMI characteristics under each diagnostic criterion. The points represent medians, while lower and upper quartiles are represented by the vertical bars.

**Figure A4:**
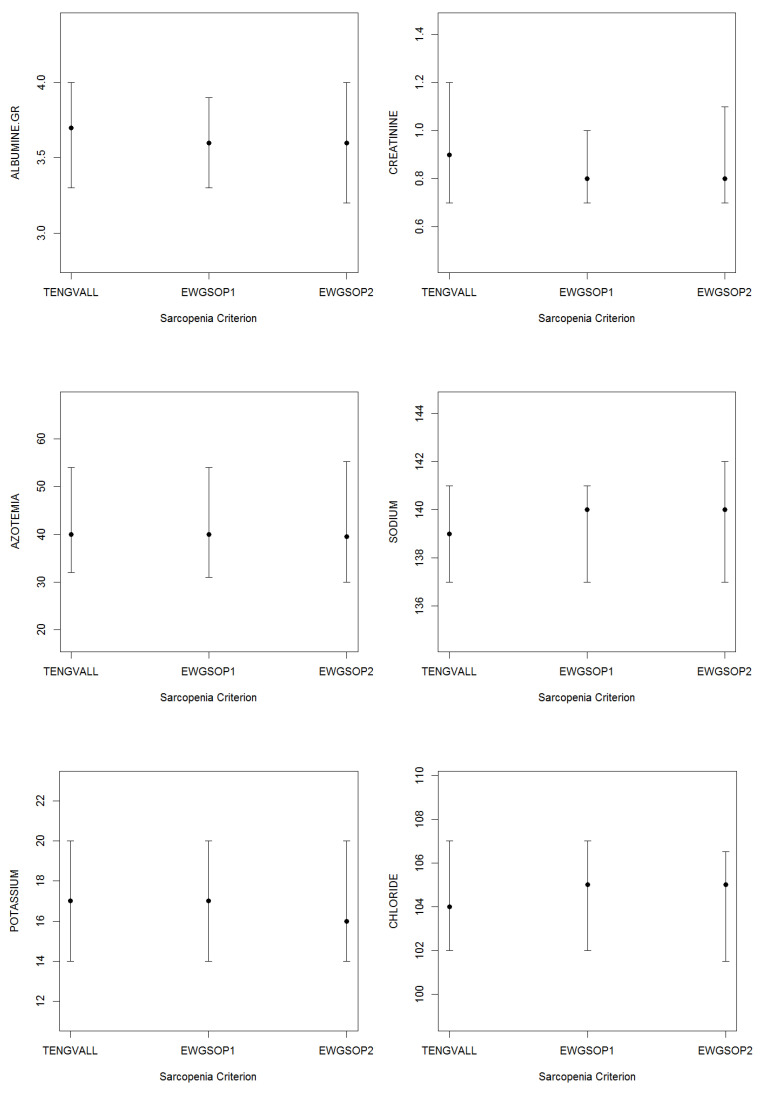
Summary of patients with low ASMI characteristics under each diagnostic criterion. The points represent medians, while lower and upper quartiles are represented by the vertical bars.

**Figure A5:**
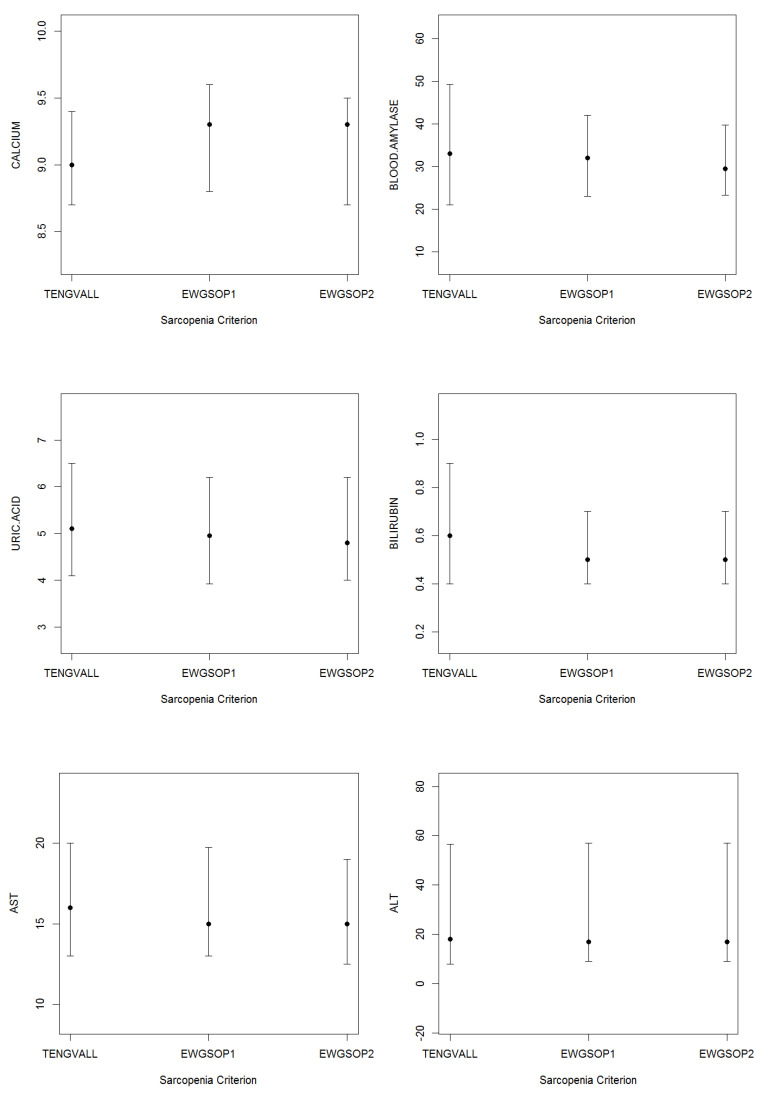
Summary for patients with low ASMI characteristics under each diagnostic criterion. The points represent medians, while lower and upper quartiles are represented by the vertical bars.

**Figure A6:**
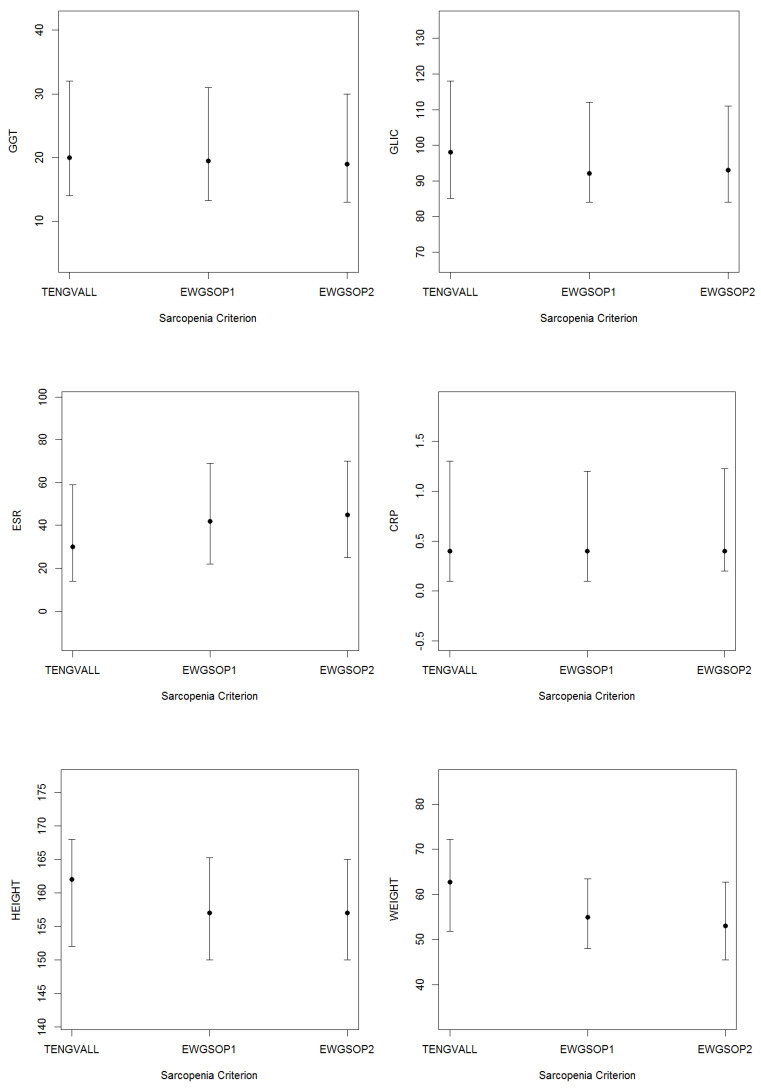
Summary for patients with low ASMI characteristics under each diagnostic criterion. The points represent medians, while lower and upper quartiles are represented by the vertical bars.

**Figure A7:**
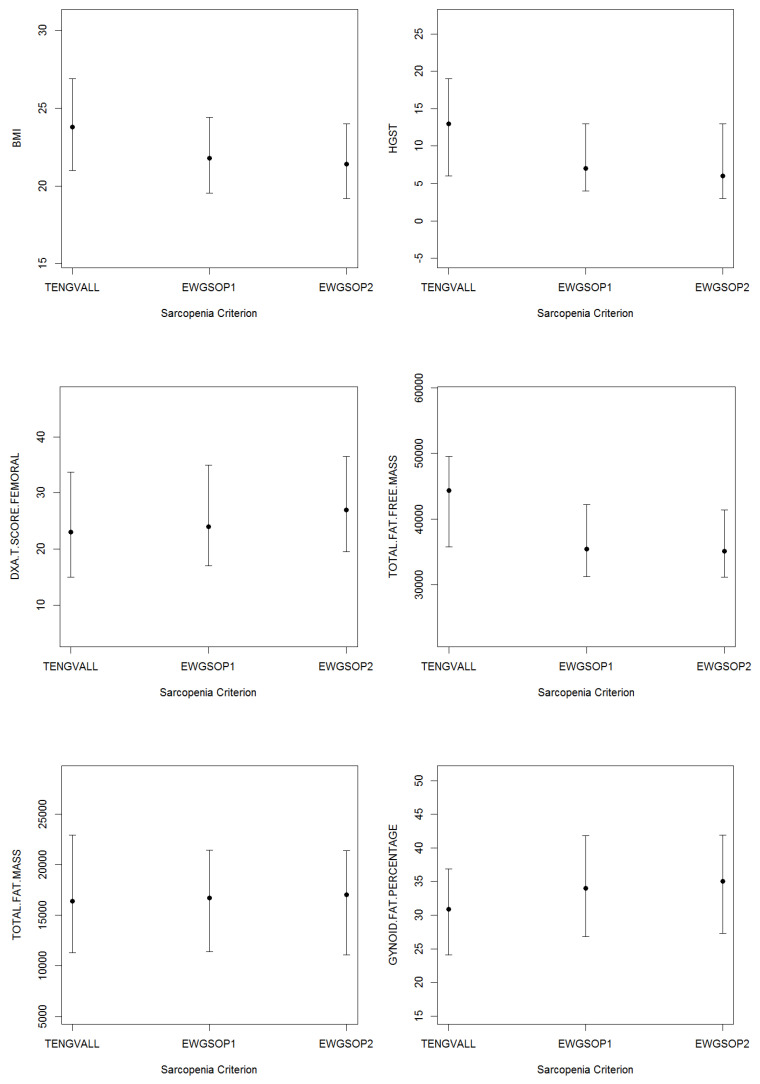
Summary for patients with low ASMI characteristics under each diagnostic criterion. The points represent medians, while lower and upper quartiles are represented by the vertical bars.

**Figure A8:**
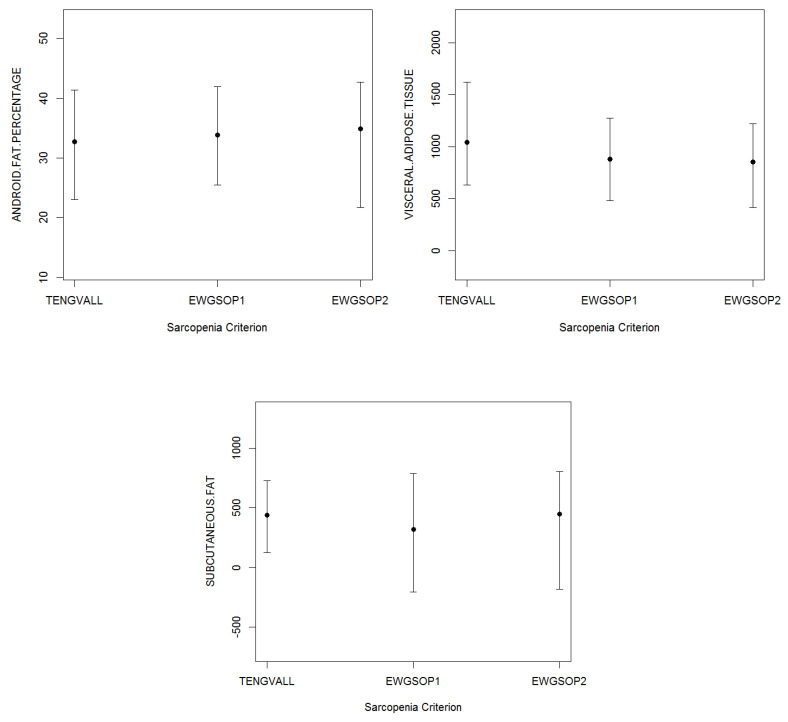
Summary for patients with low ASMI characteristics under each diagnostic criterion. The points represent medians, while lower and upper quartiles are represented by the vertical bars.

The patients’ characteristics are summarized in Table 1. The majority of patients were non-smokers with ages between 58 and 98 years. A total of 72.2% of patients were female. The great difference in our sample between the total number of men and women reflects the gender distribution of elderly people in Italy. Overall, the medians of all biomarkers were at a normal level. Further details about the characteristics of patients categorized for low ASMI are represented graphically by Figure A1, Figure A2, Figure A3, Figure A4, Figure A5, Figure A6, Figure A7 and Figure A8. 

### 3.3. Prevalence of Low ASMI

**Figure 1:**
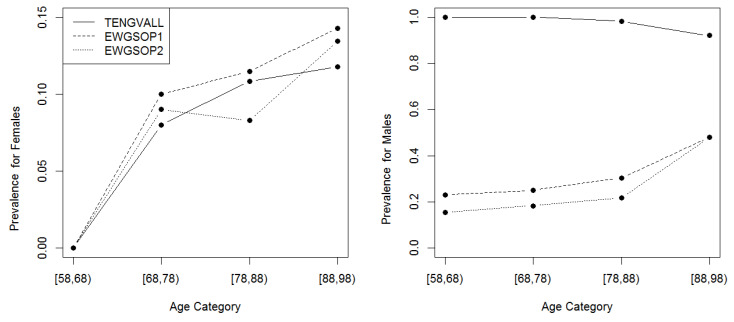
The prevalence of low appendicular skeletal muscle mass index (ASMI) under each diagnostic criterion and calculated over age categories for females and males, separately. The abbreviation used is: European working group on sarcopenia in older people 1 (EWGSOP1) and 2.

To investigate the progression of muscle loss, we used 10-year intervals represented by patients aged 58 to 68 years, 68 to 78 years, 78 to 88 years, and 88 to 98 years. Then, the prevalence of low ASMI was calculated under each diagnostic criterion for the constructed age categories. The corresponding results are depicted in Figure 1. Interestingly, for female patients, the presented plots in Figure 1 show a general consistency among the three diagnostic criteria in terms of the prevalence’s magnitude and age trend where the only minor deviation was realized over patients aged 78–88. More concretely, the prevalence of low ASMI over the first age category was negligible with an increasing trend, reaching around 15% for the last age category. 

On the other hand, the Tengvall diagnostic criterion reported a significantly higher prevalence of low ASMI (exceeding 90%), which indicates an exaggerated behavior among male patients in the study sample. Furthermore, the prevalence of low ASMI was characterized with a slight downward trend under the Tengvall diagnostic criterion. In contrast, the prevalence of low ASMI fluctuated around 20% over the first age category with a deviation of 5% between the two EWGSOP diagnostic criteria. There was an increasing trend so that the prevalence of low ASMI reached about 40% for the last age category.

**Figure 2:**
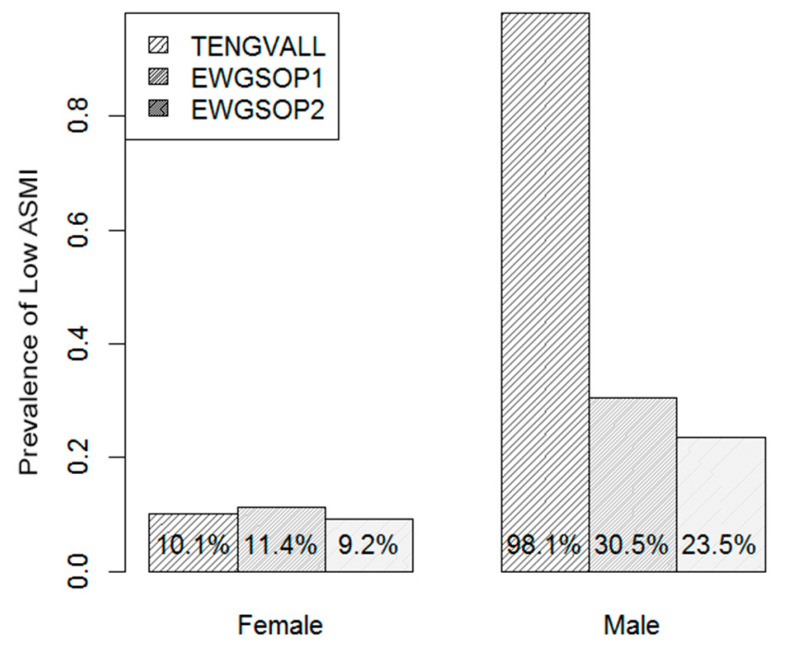
The prevalence of low ASMI (muscle loss) under each diagnostic criterion and calculated per gender.

Figure 2 shows the gender-specific prevalence of low ASMI under each diagnostic criterion. Several realizations can be derived from the presented plots. Under all diagnostic criteria, the prevalence of low ASMI for males was consistently higher than for females. Specifically, old males were more than twice as likely to be diagnosed as sarcopenic than females under the EWGSOP criteria. On the other hand, the vast majority of males in the study sample (98.1%) were diagnosed as sarcopenic under the Tengvall criterion, with a prevalence that was more than three times as high as the prevalence of low ASMI for males under the other two diagnostic criteria. The latter realization confirms an inconsistency between the Tengvall and EWGSOP diagnostic criteria for males. However, this was not the case for females; the prevalence of low ASMI for females was comparable among the three diagnostic criteria.

### 3.4. Agreement Analysis

For the sake of comparison among the three diagnostic criteria of low ASMI, the percentage of agreement along with the value of Cohen’s Kappa and the corresponding *p*-value are presented in Table 2 for females and in Table 3 for males. Overall, for females, there was sufficient agreement among all considered criteria of which EWGSOP1 and EWGSOP2 had almost perfect agreement. On the contrary, for males, the only agreement was between the EWGSOP-based criteria.

### 3.5. ASMI Loss Risk Factors

To identify the important risk factors for low ASMI under each diagnostic criterion, a two-step procedure was adopted. First, significant predictors for each criterion were identified using an univariable logistic regression. Second, the identified predictors from the first step were used to determine the best multivariable logistic regression model using the backward stepwise selection method of which predictors with a variance inflation factor of more than 10 were removed from the best selected model. The corresponding results reported for each diagnostic criterion are summarized in Table 4 for females and in Table 5 for males. It is worth mentioning that the analysis used the logistic regression model and was conducted after excluding all missing data to end up with a sample size of 201 female patients and 67 male patients. 

The overall conclusion that can be drawn from the fitted logistic regression models is that women’s height and weight played significant roles in low ASMI detection under the Tengvall criterion, while the body mass index (BMI) was identified as the main risk factor for low ASMI under the EWGSOP-based criteria.

For male patients, we noted that the small sample size hindered running the relevant logistic regression analysis. Specifically, for the Tengvall diagnostic criterion, no statistically significant predictor was identified because all male patients except one had low ASMI values. 

### 3.6. Sarcopenic Obesity

**Figure 3:**
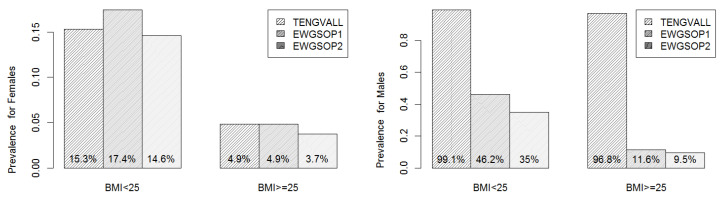
The prevalence of low ASMI under each diagnostic criterion which was calculated over BMI categories and for males and females separately.

An area of interest is the prevalence of low ASMI with respect to BMI categories. Figure 3 depicts this feature and presents the prevalence of low ASMI under each diagnostic criterion when calculated for overweight or obese patients (BMI ≥ 25 kg/m^2^) in comparison with underweight or normal patients (BMI < 25 kg/m^2^). Apparently, the prevalence of low ASMI among males is higher than that among females in both comparing groups (defined with respect to BMI).

The prevalence of low ASMI for underweight or normal females was higher than the prevalence of low ASMI for overweight or obese females (nearly three times). Moreover, the prevalence of low ASMI for females was comparable among the three diagnostic criteria. 

On the other hand, under the EWGSOP criteria, the prevalence of low ASMI for overweight or obese males was at least 35% and was notably lower than the prevalence of low ASMI for underweight or normal males (less than 12%). However, when the Tengvall criterion was considered almost all male subjects were with low ASMI. Interestingly, this exaggeration behavior of Tengvall diagnostic criterion for men was evident among both BMI-based comparing groups. 

Another concern worth investigating is the risk factors of low ASMI for overweight or obese patients. To this end, the pruned classification tree was used. It is important to note that the sample sizes for overweight or obese females and males were 108 and 37 respectively among which we had only two females diagnosed as sarcopenic, while all men were diagnosed as sarcopenic under the Tengvall criterion. 

## 4. Discussion

Low ASMI is an age-related physiological situation and, hence, it is expected to increase with age. Indeed, this expectation applies to the EWGSOP criteria, while the prevalence of low ASMI has a downward trend for the later age categories under the Tengvall criterion. Consequently, the main finding of this study was that there is no consistency between Tengvall and EWGSOP diagnostic criteria regarding the prevalence of low ASMI for males. Among men, the Tengvall equation seems to overestimate the prevalence of low ASMI (98.1% of men), versus the EWGSOP criteria (20–30%). Specifically, the Tengvall equation overestimates the prevalence of low ASMI in patients aged between 60 and 80 years old, while it aligns with the EWGSOP criteria among patients over the age of 80. However, this discrepancy does not appear for females, as the prevalence of low ASMI was comparable among the three diagnostic criteria. The overall prevalence estimate among women was similar using all the different considered criteria. According to a recent meta-analysis of 31 studies, including 9416 older Brazilians, the overall prevalence of elderly people with muscle loss was 17.0%. Prevalence was 16.0% based on low muscle mass (EWGSOP criterion) and 17.0% based only on low muscle mass (evaluated by DXA and Baumgartner’s criteria) [22].

An interesting observation was made by stratifying subjects with BMI values over and under 25 kg/m^2^. We found that the percentage of ASMI loss decreased by more than double when using the EWGSOP1 and EWGSOP2 criteria. BIA is known to underestimate fat mass and overestimate muscle mass [11]. Previous studies found that the BIA-based prevalence of low ASMI was higher than that determined by the DXA-based approach [23,24]. Moreover, the use of BIA for muscle mass assessment presents some drawbacks, mainly due to hydration problems usually observed in older persons, possibly resulting in an underestimation of body fat and an overestimation of low ASMI [25]. A previous study, as a bedside alternative to DXA, recommended the use of BIA instead of anthropometry for discriminating between low and normal muscle mass [26]. When BIA is used, Tengvall, together with Kyle and Sergi, is a recommended method because of its accuracy for both men and women [27]. Although BIA presents some limitations, it is inexpensive and worth using in the clinical practice routine.

To the best of our knowledge, this is the first study in the literature to compare EWGSOP1 and EWGSOP2 with Tengvall as different criteria for low ASMI prevalence. Nevertheless, we think that further investigations are needed to better evaluate the use of BIA in the diagnosis of ASMI loss.

Secondarily, our analysis aimed to investigate which metabolic and other risk factors are associated with a poor ASMI as diagnosed using different criteria. In that regard, sarcopenic subjects identified using the EWGSOP criteria were associated with a reduction in BMI. The fact that BMI was lower in patients with low ASMI was recently confirmed by Fonseca et al. [28], who evaluated 168 male subjects characterized by different weights and heights, in addition to observations reported in the literature [29,30]. In our study, patients with low ASMI, according to the Tengvall criteria, were associated with a larger number of risk factors. For instance, they showed a decrease in BMI, cholesterol, and platelet levels, and belonged to a lower socioeconomic group. A recent meta-analysis involving 62,273 individuals with poor skeletal muscle index revealed that there are nine metabolic risk factors (i.e., BMI, fasting glucose, systolic and diastolic blood pressure, homeostasis model assessment to quantify IR (HOMA-IR), triglycerides, total cholesterol, high density lipoprotein (HDL), and low density lipoprotein (LDL)) [31].

Among metabolic markers, our analysis showed that albumin values were lower in patients considered to have a low ASMI according to the EWGSOP criteria and higher in subjects with low ASMI screened by the Tengvall Equation. These results confirm the fact that the EWGSOP criterion is pertinent and better detects a loss of muscle mass and its related risk factors. In fact, albumin is an important marker of malnutrition. Uemura et al. [32] confirmed that sarcopenia and low serum albumin level together may increase the risk of incident disability in older adults. For this reason, we strongly encourage the implementation of nutritional assessment in older adults in order to detect early conditions like sarcopenia and malnutrition. Moreover, as recently suggested by Gonzalez et al. [33], the terms and cutoff values have been suggested in the international consensus of cancer cachexia.

The main limitation of this study is its use of a single-frequency (50 kHz) BIA device (Akern BIA 101, Bioresearch, Florence). As demonstrated by a recent research on sarcopenia evaluation, the use of eight electrode MF BIA validated an accurate equation to estimate ALM using theoretical age-independent models, which fit better with the established ASMI cutoff values. Yamada et al. [26] reported that if the manufacturer’s own undisclosed formula is used, different BIA devices provide different SMI values. However, if one disclosed equation is used, even with different devices, MF-BIAs provide the same SMI values over a wide age range (18 to 89 years) [34].

In addition, when using a single-frequency BIA device, evaluation is affected by edema or inter-segmental water shift, particularly in peripheral segments, such as the ankle and wrist. If a person has edema, in particular peripheral edema, the BIA overestimates the actual ALM. The opposite occurs if patients have dehydration; the ALM will be underestimated. To solve this problem, previous studies have used proximal electrode placement locations using multi frequency BIA, which provides a more accurate evaluation. The issue is not mainly related to the Tengvall formula, but rather to the device that we used for applying this formula [35].

## 5. Conclusions

In conclusion, muscle loss is an age-related syndrome as shown by all criteria, and thus it is expected to increase with age. Indeed, this expectation applies to the EWGSOP criteria, while the prevalence of low ASMI has a trend for the later age categories under the Tengvall criterion. Regarding gender, the prevalence of males with a low ASMI is apparently higher than that of females. Moreover, in females, the prevalence is comparable among the three diagnostic criteria, while in males, it is significantly higher under Tengvall than the other two criteria. 

## Figures and Tables

**Table 1:** The patients’ characteristics.

Characteristics	Total (*n* = 765)		Male (*n* = 213, 27.8%)		Female (*n* = 552, 72.2%)	
Smoke: Yes/No	53/518		14/142		39/376	
Age (years)	82.0	(8.2)	80.0	(8.0)	82.0	(9.0)
BI (score)	70.0	(35.0)	68.0	(37.0)	71.0	(35.0)
WBC (K/µL)	6.6	(2.5)	6.8	(2.7)	6.5	(2.5)
RBC (M/µL)	4.2	(0.7)	4.3	(0.7)	4.2	(0.7)
HB (g/dL)	30.0	(22.0)	33.0	(23.0)	29.0	(21.5)
HCT (%)	38.2	(5.7)	39.4	(6.2)	37.9	(5.4)
PLT (K/µL)	236.8	(97.1)	217.5	(103.5)	242.0	(94.1)
Iron (µg/dL)	66.0	(43.0)	66.0	(40.0)	67.0	(43.0)
Triglyceride (mmol/L)	106.0	(58.0)	99.5	(47.2)	110.0	(62.0)
Cholesterol (mmol/L)	182.0	(62.0)	161.0	(50.0)	190.0	(60.0)
HDL (mmol/L)	49.0	(19.0)	44.0	(15.0)	51.0	(19.0)
LDL (mmol/L)	108.0	(48.2)	96.2	(46.2)	113.0	(49.3)
Total Proteins (g/dL)	6.6	(0.8)	6.6	(0.7)	6.6	(0.8)
Albumin (%)	56.5	(6.2)	55.7	(7.5)	56.6	(5.9)
Albumin (g/dL)	3.7	(0.6)	3.7	(0.7)	3.8	(0.6)
Creatinine (mg/dL)	0.8	(0.3)	0.9	(0.5)	0.8	(0.3)
Azotemia (mg/dL)	40.0	(21.0)	41.0	(22.0)	40.0	(22.0)
Sodium (mmol/L)	140.0	(4.0)	139.0	(4.0)	140.0	(4.0)
Potassium (mmol/L)	16.0	(6.0)	17.0	(6.0)	16.0	(5.0)
Chloride (mmol/L)	104.0	(5.0)	104.0	(5.0)	104.0	(5.0)
Calcium (mmol/L)	9.1	(0.7)	9.1	(0.7)	9.1	(0.7)
Blood Amylase (U/L)	32.0	(30.8)	33.0	(28.5)	31.0	(31.0)
Uric Acid (mg/dL)	4.9	(2.1)	5.4	(2.3)	4.8	(1.9)
Bilirubin (mg/dL)	0.6	(0.4)	0.6	(0.5)	0.6	(0.4)
AST (IU/L)	17.0	(8.0)	16.0	(7.2)	17.0	(7.0)
ALT (IU/L)	17.0	(32.0)	18.0	(46.2)	17.0	(30.0)
GGT (U/L)	19.0	(17.0)	20.5	(20.2)	19.0	(16.0)
GLIC (mg/dL)	96.0	(33.5)	98.5	(33.0)	95.0	(35.0)
ESR (mm/h)	32.0	(36.0)	28.0	(46.0)	33.0	(33.0)
CRP (mg/dL)	0.3	(0.7)	0.4	(1.4)	0.2	(0.6)
Height (cm)	154.0	(12.0)	165.0	(10.0)	152.0	(8.0)
Weight (kg)	59.9	(17.3)	66.2	(17.0)	57.3	(17.0)
BMI (kg/m^2^)	24.6	(6.2)	24.4	(5.9)	24.6	(6.7)
HGST (kg)	9.0	(10.0)	15.0	(12.5)	8.0	(8.8)
Femoral T-Score (DXA)	24.0	(17.0)	20.0	(16.8)	25.0	(16.0)
Total Fat Mass (g)	19,830.0	(13,258.2)	17,051.0	(11,221.0)	20,576.0	(13,775.8)
Gynoid Fat (%)	38.9	(16.0)	28.8	(10.8)	42.4	(12.8)
Android Fat (%)	37.8	(20.4)	31.9	(17.5)	39.9	(20.3)
Visceral Adipose Tissue (g)	888.5	(871.7)	1199.1	(971.9)	785.3	(735.0)
Subcutaneous Fat (g)	698.5	(938.7)	441.8	(553.2)	858.9	(987.2)

Categorical variables are represented as ratios. Numerical variables are summarized by the median and (inter-quartile range). All the presented summary statistics have been calculated after excluding the missing data. The abbreviations used are: Barthel Index (BI); white blood cells (WBC); red blood cells (RBC); hemoglobin (HB); hematocrit (HCT); platelets (PLT); high density lipoproteins (HDL); low density lipoproteins (LDL); aspartate aminotransferase test (AST); alanine aminotransferase test (ALT); gamma-glutamyl transferase (GGT); glycaemia (GLIC); eritrocyte sedimentation rate (ESR); C-reactive protein (CRP); body mass index (BMI); hand grip strength test (HGST); and Dual X-ray Absortiometry (DXA).

**Table 2:** The agreement analysis among the diagnostic criteria for low ASMI in females.

Diagnostic Criteria	TENGVALL	EWGSOP1
EWGSOP1	84.6%, 0.200 (<0.001)	
EWGSOP2	86.4%, 0.224 (<0.001)	97.8%, 0.883 (<0.001)

The reported figures represent the percentage of agreement and the Cohen’s Kappa with the corresponding *p*-value in parentheses. EWGSOP: European working group on sarcopenia in older people.

**Table 3:** The agreement analysis among the diagnostic criteria for low ASMI in males.

Diagnostic Criteria	TENGVALL	EWGSOP1
EWGSOP1	32.4%, 0.017 (0.181)	
EWGSOP2	25.4%, 0.012 (0.263)	93.0%, 0.822 (<0.001)

The reported figures represent the percentage of agreement and the Cohen’s Kappa with the corresponding *p*-value in parentheses.

**Table 4:** The results of logistic regression for risk factors associated with low ASMI among females.

Predictors	TENGVALL		EWGSOP1		EWGSOP2	
Azotemia	−0.0780	(0.0316) *			−0.0512	(0.0423) *
Height	−0.1634	(0.0277) *				
Weight	−0.1931	(0.0002) *	+0.1337	(0.0232) *	+0.1130	(0.0880)
BMI			−0.3610	(0.0077) *	−0.3362	(0.0278) *

The reported figures represent the log-odds ratios (*p*-values) where the asterisk indicates significance at 5% level. Note that each column represents the results of the fitted logistic regression model under a specific diagnostic criterion.

**Table 5:** The results of logistic regression for risk factors associated with low ASMI among males.

Predictors	TENGVALL	EWGSOP1		EWGSOP2	
ESR				+0.0818	(0.0130) *
Height					
Weight					
BMI		−0.2598	(0.0373) *		
HGST		−0.0691	(0.1560)		
Femoral T-Score		+0.0507	(0.0373) *	+0.1377	(0.0056) *

The reported figures represent the log-odds ratios (*p*-values) where the asterisk indicates significance at 5% level. Note that each column represents the results of the fitted logistic regression model under a specific diagnostic criterion.

## Appendix A

The following plots (from Figure A1, Figure A2, Figure A3, Figure A4, Figure A5, Figure A6, Figure A7 and Figure A8) summarize the 41 predictors for patients with low ASMI under each diagnostic criterion.
